# Evaluation of the Total Design Method in a survey of Japanese dentists

**DOI:** 10.1186/1471-2288-5-27

**Published:** 2005-08-23

**Authors:** Yukie Nakai, Peter Milgrom, Toshiko Yoshida, Chikako Ishihara, Tsutomu Shimono

**Affiliations:** 1 Department of Behavioral Pediatric Dentistry, Okayama University Graduate School of Medicine, Dentistry and Pharmaceutical Sciences, Okayama, Japan; 2 Department of Dental Public Heath Sciences, University of Washington, Seattle, USA

## Abstract

**Background:**

This study assessed the application of the Total Design Method (TDM) in a mail survey of Japanese dentists. The TDM was chosen because survey response rates in Japan are unacceptably low and the TDM had previously been used in a general population survey.

**Methods:**

Four hundred and seventy eight dentist members of the Okayama Medical and Dental Practitioner's Association were surveyed. The nine-page, 27-item questionnaire covered dentist job satisfaction, physical practice, and dentist and patient characteristics. Respondents to the first mailing or the one-week follow-up postcard were defined as early responders; others who responded were late responders. Responder bias was assessed by examining age, gender and training.

**Results:**

The overall response rate was 46.7% (223/478). The response rates by follow-up mailing were, 18% after the first mailing, 35.4% after the follow-up postcard, 42.3% after the second mailing, and 46.7% after the third mailing. Respondents did not differ from non-respondents in age or gender, nor were there differences between early and late responders.

**Conclusion:**

The application of TDM in this survey of Japanese dentists produced lower rates of response than expected from previous Japanese and US studies.

## Background

Mail survey questionnaires of dentists as well as the general public have been used widely in the U.S. and response rates are generally high. In contrast the use of mail surveys in Japan has been less successful. Japanese textbooks on social science research techniques report return rates of no more than 20–40% [1-3]. A mail survey conducted by one of the local Japanese dental associations had a response rate of 10% (unpublished data). Mail surveys reported in the Japanese medical literature had response rates ranging from 49 to 90% [4-8]. Research subjects in the various studies were the physicians and residents working at two private University hospitals (Response rate 49.1%) [4], the institutions belonging to an oncology group (Response rate 90.2%) [5], the council members of the Japanese society of child neurology (Response rate 72.8%) [6], ophthalmologists in hospitals and clinics (Response rate 73%) [7], and psychologists (Response rate not given) [8]. However, the publications lack methodological detail. Only two of the five, for example, provide the source of the mailing lists. In two of the surveys, questionnaires were sent to a representative at each hospital or institution rather than to individuals directly [4,5]. None of the five papers indicated whether the studies were sponsored by a professional association, or university or other group. One of five publications indicated that an advance letter was sent before the questionnaire [7]. Only one paper specified whether participants were told how the data would be used [7]. None of the papers explained whether an incentive was included in the mailing of the questionnaire. Other details generally missing were the length of the questionnaire (missing in 2/5) [4,6,8], telephone contacts for more information (missing in all 5) or assurance of confidentiality (missing in all 5).

The Total Design Method (TDM), which was developed by Dillman and includes personalization of the cover letter and repeated follow-ups, was designed to achieve high response rates and minimize the potential influence of systemic nonresponse bias [9]. The response rate generally is lower in surveys of the general public and higher in surveys of professionals although this varies by group and subject. Locker and colleagues reported a 71.6% response rate when an oral health questionnaire using the TDM was used to survey the general population from voters' lists [10]. Fiset and colleagues mailed questionnaires concerning dental malpractice claims to dentists using the TDM, and reported a 69.6% response rate [11].

In the only application of the TDM in Japan to date, Jussaume and colleagues reported a 55.6% response rate for a survey of the general population on the subject of 748 when those surveyed were selected from telephone listings [12].

No work has been done on adapting the TDM to Japanese dental populations. The aim of this study was to assess the application of the TDM in a mail survey of Japanese dentists.

## Methods

### Subjects

The questionnaire was mailed to all 482 dentist members on Okayama Medical and Dental Practitioner's Association list. Out of 482 questionnaires sent out, four dentists were excluded because they had closed their office due to sickness or had shared replying survey with a spouse dentist. The final survey population was 478 dentists. Potential subjects were informed in the cover letter that participation in the study was voluntary and that individual responses would be confidential.

### Questionnaire development

**Figure 1:**
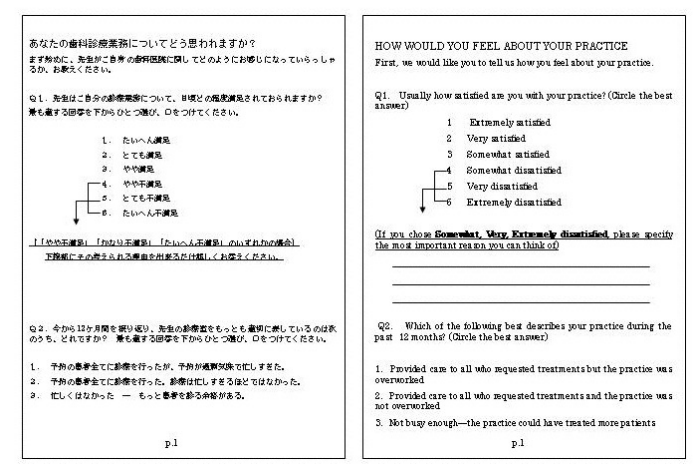
The first page of the questionnaire and its English translation.

A nine-page, 27- item questionnaire was designed in English using questions derived from earlier surveys. It covered four categories: 1) dentist job satisfaction, 2) physical practice, 3) dentist and 4) patient characteristics. Instrumentation was translated from English to Japanese by a native speaker, and then back-translated by another native speaker to ensure comparability to the original English form (see Figure 1). The questionnaire booklet was organized so that easier and less personal questions were asked initially and more difficult or personal questions were asked at the end of the questionnaire. The questionnaire was pretested among the alumni practicing out of Okayama prefecture before use. The questionnaire was formatted into a 182 × 257 mm booklet style to make it appear easier and less time-consuming to complete.

### Procedures

The Okayama Medical and Dental Practitioner's Association agreed to participate and endorse the study.

The procedures followed were generally those recommended by Jussaume and Yamada [12] who had previously adapted the TDM to Japan. In designing the letters, a strong emphasis was placed on three essential features of the TDM. First, respondents were told how their names were selected, that their responses would represent those of many other Japanese dentists, and that their participation was invaluable. Second, the confidentiality of the survey was emphasized and participants were promised that their names would never be placed on the questionnaire. Finally, as an incentive for participation, a decision was made with the Association that respondents would be offered a report of the results of this study. No personal incentive was included in the survey because Japanese culture values service to the group rather than the individual [12].

**Figure 2:**
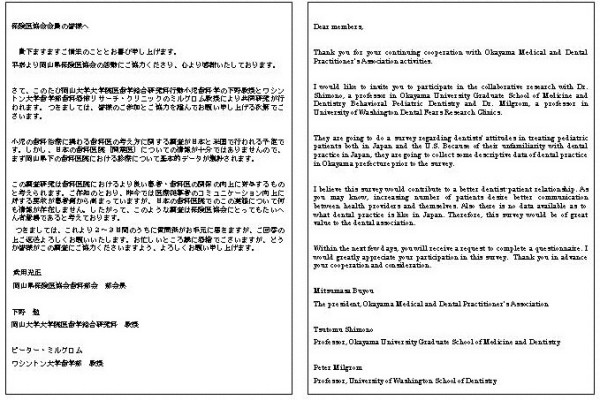
Advance letter.

**Figure 3:**
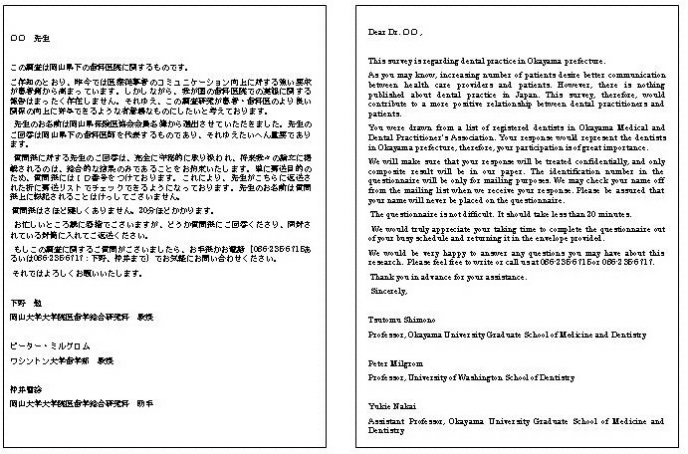
Initial letter sent in the first mailing.

**Figure 4:**
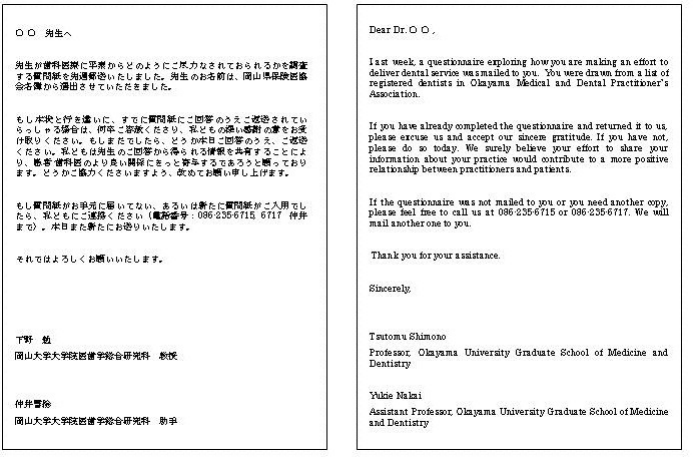
Follow-up card.

**Figure 5:**
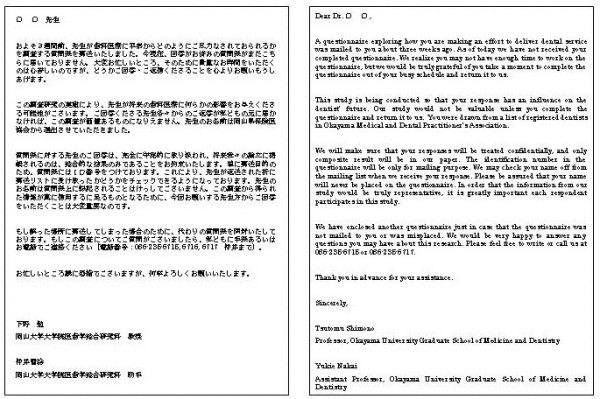
Second letter sent in the second mailing.

**Figure 6:**
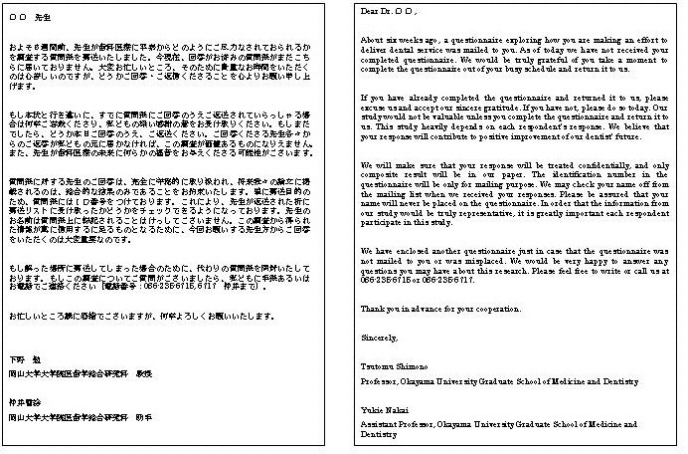
Third letter sent in the third mailing.

Approximately one week before the first mailing of the questionnaire, an advance letter including the Association endorsement was sent to all the dentists introducing the researchers and explaining the importance of the study (see Figure 2). The letters were not personalized and not individually signed. The letter noted that the participant would receive the questionnaire in a couple of days. The envelopes were personally addressed and stamped. In the first questionnaire mailing, the participants received a letter again explaining the importance of the study and assuring confidentiality (see Figure 3), the questionnaire booklet, and a stamped self addressed return envelope. Identification number markers were used on questionnaires so that respondents could be checked off the mailing list. A follow-up postcard (see Figure 4), encouraging participation, was sent about one week later. Three weeks after the first mailing, a replacement questionnaire, a stamped return envelope and a cover letter (see Figure 5) were sent to any dentists who had not responded. Dentists who did not respond within six weeks after the original mailing received a cover letter (see Figure 6), a second replacement questionnaire, and a stamped self addressed return envelope.

Japanese standard number 3 size (235 × 120 mm) envelopes of an light yellow green color were used. Addresses were written on envelopes from left to right in the manner of most Japanese business correspondence.

### Data handling and analysis

The data from questionnaires received within four months of the first mailing were entered into a database in Excel 2000 (Microsoft), and were checked for accuracy. Data management and analyses were conducted using SPSS version 11.5.

A two-pronged strategy was used to assess bias. First, age and gender of respondents and non-respondents, provided by the association list, were compared. Second, we compared study variables for early and late respondents. Respondents to the first mailing or the one-week follow-up postcard were defined as early responders; others who responded were late responders. Study variables included age, gender, years in practice, practice satisfaction, practice status, practice location, patient number seen per day, having postgraduate training, total hours of continuing dental education taken for the past 12 months, employment status (owner vs. non-owner), number of practice locations, yearly gross income before any expenses or taxes. T-tests, Fisher's exact test and Chi-square analyses were used to compare differences between the groups to assess respondent representativeness.

## Results

### Response rate

The overall response rate was 46.7% (223/478). The cumulative response rates by each follow-up mailing are shown in Table 1. Ten dentists declined to participate. The primary reason given for refusal was that the dentist was not comfortable in answering personal questions.

**Table 1:** Cumulative response rate to Japanese dental questionnaire using the TDM

	**1^st ^Mailing**	**Follow-up Card**	**2^nd ^Mailing**	**3^rd ^Mailing**
Response rate	18.0 %	21.2%	10.7%	7.6%
	(86/478)	(83/392)	(33/309)	(21/276)
Cumulative		35.4%	42.3%	46.7%
response rate		(169/478)	(202/478)	(223/478)

### Respondent representativeness

Respondents did not differ from non-respondents for the gender and age, nor were there differences between early and late responders for any of the 12 of variables that were compared except that the late responders have taken less hours of continuing dental education during the past 12 months (17.5 vs. 29.2 hours; t = 1.95, p = 0.05) (Table 2). There was a trend for late responders to be more likely to have received postgraduate training (27.3% vs. 16.3%; Fisher's exact test, p = 0.08)

**Table 2:** Responses on study variable for early/late responders

**Variables**	**N**	**Early responders**	**Late Responders**	**p**
Mean age (SD)	223	45.6 (9.8)	45.6 (10.9)	NS
% female	223	9.5	7.4	NS
Mean months in practice (SD)	220	163 (117)	172 (129)	NS
Practice satisfaction (% dissatisfied)	215	24.7	22.6	NS
Busyness (%not busy enough)	220	28.7	30.2	NS
Practice location (% patients from rural areas)	212	28.7	29.2	NS
Patient visits/day (mean, SD)	212	34.5 (25.6)	32.2 (26.2)	NS
Postgraduate training (%no)	215	25.5	15.1	0.08
Total hrs CDE/12 mos (Mean, SD)	197	29.2 (58.7)	17.5 (24.9)	0.05
Employment status (% non owner)	219	15.7	13.2	NS
>1 practice location	214	4.9	7.8	NS
				
Annual gross income before expense/taxes (% less than ¥30,000,000	194	23.0	34.8	NS
Year of graduation (mean, SD)	212	1981 (10.7)	1981 (11.7)	NS

## Discussion

The TDM, as generally adapted by Jussaume and Yamada [12], was used in a survey of Japanese dentists. Previously Jussaume and Yamada obtained nearly identical response rates when they surveyed the general public in Japan (55.6%) and the U.S. (57.5%) using this method. The application of the TDM in this survey of dental practice produced a lower response rate (46.6%) than expected but with little response bias. The results of a low response rate (43%) without non-response bias was previously reported in the US dentist population [13] although other studies using this method have produced higher response rates. Dentists can be considered to have sufficiently similar education, income, and interest to be considered a homogeneous group. If there is little difference between the respondents and non-respondents, a smaller percentage of return might be acceptable. Parashos and colleagues, who reported a dentist survey in Australia and New Zealand also using the TDM, found significant differences between early and late respondents in responses to a specific survey question of topical interest despite the absence of differences in the demographic data [14]. They emphasize the importance of using methods to achieve a high response rate to overcome such bias.

The lower than anticipated response rate of this study may have resulted from our failure to follow all aspects of the TDM fully. One of the differences found in procedures between Jussaume's [12] and this study was that neither *Inkan *(personal seal) nor signature was used to the letter in this study. Jussaume said that it could convey to the respondent the importance which researchers placed on the project. The other difference was that the letter was not written in longhand in this study. Japanese respondents are hypothesized to react more positively to a survey seeing the effort taken to write out their names in longhand. Japanese dentists may also be more reluctant to answer the questions that they feel too personal. Ten dentists had refused to participate in this study due to such reasons. One of the authors asked the primary reasons why they didn't participate to another five dentists. Two of these five dentists said the questions were too personal and that there were too many questions to answer. One dentist said that he didn't want his practice to be compared with others. Two said that topic was not interesting enough to make them want to participate.

The response rate was, however, much greater than that of another unpublished survey of Japanese dentists that achieved a 10 % response rate. The follow-up contact and repeated mailing to non-respondents increased our sample size by more than a quarter (28.7%). Before using the postcard reminder, the response rate was one-third of the final rate, suggesting that follow-up contact is critical to bolstering mail survey response rates. This is consistent with research indicating that follow-up contact has the most positive effect on return rates [11,15-17].

Our results are encouraging, and demonstrate the feasibility of using TDM to study a population of Japanese dentists.

## Conclusion

The application of TDM in this survey of Japanese dentists produced lower rates of response than expected from previous Japanese and US studies with little response bias.

## List of abbreviations

TDM Total Design Method

## Competing interests

The author(s) declare that they have no competing interests.

## Authors' contributions

YN participated in the design of the study, made the instruments (translated English version to Japanese), negotiated with Okayama Medical and Dental Practitioner's Association to be given endorsement, collected the data, performed the statistical analyses, and drafted the manuscript. PM participated in the design of the study and in writing the manuscript. TY participated in the design of the study and developed the English version of the instruments for this paper. CI collected the data and performed the statistical analyses. TS participated in the design of the study. All authors read and approved the final manuscript.

## Pre-publication history

The pre-publication history for this paper can be accessed here:


